# “Calcium bombs” as harbingers of synaptic pathology and their mitigation by magnesium at murine neuromuscular junctions

**DOI:** 10.3389/fnmol.2022.937974

**Published:** 2022-07-26

**Authors:** Kosala N. Dissanayake, Robert R. Redman, Harry Mackenzie, Michael Eddleston, Richard R. Ribchester

**Affiliations:** ^1^Euan MacDonald Centre for Motor Neurone Disease Research, The University of Edinburgh, Edinburgh, United Kingdom; ^2^Centre for Discovery Brain Sciences, The University of Edinburgh, Edinburgh, United Kingdom; ^3^Clinical Pharmacology, Toxicology and Therapeutics, Centre for Cardiovascular Science, Queen’s Medical Research Institute, The University of Edinburgh, Edinburgh, United Kingdom

**Keywords:** neuromuscular junction, muscle contraction, synapse, synaptic pathology, ALS, calcium imaging, organ culture

## Abstract

Excitotoxicity is thought to be an important factor in the onset and progression of amyotrophic lateral sclerosis (ALS). Evidence from human and animal studies also indicates that early signs of ALS include degeneration of motor nerve terminals at neuromuscular junctions (NMJs), before degeneration of motor neuron cell bodies. Here we used a model of excitotoxicity at NMJs in isolated mouse muscle, utilizing the organophosphorus (OP) compound omethoate, which inhibits acetylcholinesterase activity. Acute exposure to omethoate (100 μM) induced prolonged motor endplate contractures in response to brief tetanic nerve stimulation at 20–50 Hz. In some muscle fibers, Fluo-4 fluorescence showed association of these contractures with explosive increases in Ca^2+^ (“calcium bombs”) localized to motor endplates. Calcium bombs were strongly and selectively mitigated by increasing Mg^2+^ concentration in the bathing medium from 1 to 5 mM. Overnight culture of nerve-muscle preparations from *Wld^S^* mice in omethoate or other OP insecticide components and their metabolites (dimethoate, cyclohexanone, and cyclohexanol) induced degeneration of NMJs. This degeneration was also strongly mitigated by increasing [Mg^2+^] from 1 to 5 mM. Thus, equivalent increases in extracellular [Mg^2+^] mitigated both post-synaptic calcium bombs and degeneration of NMJs. The data support a link between Ca^2+^ and excitotoxicity at NMJs and suggest that elevating extracellular [Mg^2+^] could be an effective intervention in treatment of synaptic pathology induced by excitotoxic triggers.

## Introduction

The defining characteristic of amyotrophic lateral sclerosis (ALS) is progressive neuromuscular paralysis, associated with degeneration of cortical (“upper”) and spinal (“lower”) motor neurons and their axonal and synaptic connections with skeletal muscle fibers (Cleveland and Rothstein, 2001). Familial or genetic factors are implicated in 5–10% of patients diagnosed with ALS, while the majority are diagnosed with sporadic forms of the disease. A minority of ALS patients also show evidence of frontotemporal dementia (FTD) with progressive impairment of cognitive function (Abramzon et al., 2020), suggesting that ALS/FTD represents a spectrum of disease (van Es et al., 2017). Nuclear translation and cytoplasmic aggregation of TDP43 protein in motor neuron cell bodies (Mackenzie and Rademakers, 2008), or mutations and haploinsufficiency in expression of *C9ORF72* (Balendra and Isaacs, 2018), have been implicated in several forms of ALS and FTD. However, there is no ubiquitous association with any specific risk factor or molecular mechanism that forms a basis for a unifying theory of ALS/FTD (Keon et al., 2021). Excitotoxicity has been hypothesized to trigger several forms of neurodegenerative disease, and it has long been considered a potential explanation for ALS, as well as providing a rationale for its mitigation by riluzole or edavorone, presently the only fully licensed treatments for ALS (Cheah et al., 2010; Brooks et al., 2022; Sever et al., 2022; Tarantino et al., 2022). However, the potential associations between activity or excitotoxicity with ALS or FTD remain controversial (Chiò et al., 2005; Huisman et al., 2013; Gallo et al., 2016; Starr and Sattler, 2018).

Abnormal function and degeneration of neuromuscular synapses are among the earliest signs of ALS, in humans and in animal models, and these signs evidently occur before overt loss of motor neurons (Fischer et al., 2004; Schaefer et al., 2005; David et al., 2007; Martineau et al., 2018). In some instances, exposure to toxins acting on cholinergic synapses, including neuromuscular junctions (NMJs) carries an increased risk of ALS. For instance, inhibitors of acetylcholinesterase (anti-AChEs), which prolong cholinergic synaptic depolarization, have been implicated in the increased incidence of ALS in military personnel and veterans diagnosed with “Gulf War Syndrome” (Haley, 2003; Kasarskis et al., 2009). NMJ dysfunction, paralysis and degeneration also occur following acute exposure to agricultural insecticides containing organophosphorus (OP) anti-AChE compounds dissolved in organic solvents, with possible links to ALS (Eddleston et al., 2012; Pamphlett, 2012; Merwin et al., 2017; Dissanayake et al., 2021a,b). Acute exposure of rodent muscles to OP anti-AChEs has a number of pathophysiological effects and consequences, including hypercontraction of muscle fibers in the region of their NMJs, and this has been linked to subsequent development of a focal Ca^2+^-dependent myopathy, and associated in some studies with degeneration of motor nerve terminals (Leonard and Salpeter, 1979; Duxson and Vrbová, 1985; Meshul et al., 1985; Ferry and Cullen, 1991; Zhu et al., 2014). However, experimental evidence directly linking excitotoxic neuromuscular activity, Ca^2+^ levels, focal hypercontraction of motor endplates, and synaptic degeneration is incomplete.

Our aim in the present study was to establish whether Ca^2+^ stress at NMJs, induced by prolonged endplate depolarization, could be linked to their subsequent degeneration. We found, using Fluo-4 as a Ca^2+^ indicator, that repetitive stimulation when AChE enzymic activity was inhibited gave rise to explosive, prolonged increases in post-synaptic [Ca^2+^], localized to motor endplates. These “calcium bombs” were mitigated by modest increases on the extracellular concentration of Mg^2+^ (from 1 to 5 mM). Inhibiting AChE with OP compounds also accelerated synaptic degeneration at NMJs in organ cultures of isolated nerve-muscle preparations. Remarkably, synaptic degeneration was also substantially reduced when extracellular Mg^2+^ was increased from 1 to 5 mM. The data strengthen the link between Ca^2+^ excitotoxicity and synaptic pathology and suggest it may be worthwhile to re-evaluate potential mitigating effects of Mg^2+^ therapy where synapses are vulnerable to excitotoxicity, perhaps including relevant forms of ALS/FTD.

## Materials and methods

### Animals and tissues

Experiments were carried out on isolated tissues from adult (age 1–6 months) wild-type C57Bl6 and C57BlWld*^S^* mice, bred and maintained in University of Edinburgh animal care facilities, under standard conditions closely monitored by appointed Veterinary Officers and regularly inspected under institutional license by the UK Home Office. C57Bl-*Wld^S^* mice were backcrossed with the transgenic line *thy1.2YFP16*, to obtain double homozygotes (Feng et al., 2000; Wong et al., 2009). Mice of both sexes were killed by isoflurane anesthetic overdose (>5% in air) and cervical dislocation, in accordance with approved UK Home Office Schedule 1. Flexor digitorum brevis (FDB), lumbrical (DL), or triangularis sterni (TS) muscles and their respective tibial nerve or intercostal nerve supplies were promptly dissected and maintained in mammalian physiological saline (MPS) with the following composition (mM): Na^+^ (158); K^+^ (5); Ca^2+^ (2); Mg^2+^ (1); Cl^–^ (169); glucose (5); HEPES (5); pH 7.2–7.4. Solutions were bubbled with air for at least 20 min. Most experiments were conducted at room temperature (19–25°C). Drugs were added directly from aqueous stock solutions to bathing solutions to give the required concentrations. Aliquots (10–100 μl) were either pipetted directly into the recording chamber and thoroughly mixed with MPS, or solutions containing the required final concentration in 50 ml volumes were rapidly exchanged with the solution in the recording chamber (volume approximately 10 ml) using coupled back-to-back 20–50 ml syringes connected to ports at opposite ends of the chamber. Baffles built into the chamber facilitated laminar flow and complete solution exchange within 10–20 s.

### Drugs and toxins

Omethoate and dimethoate were obtained from Toronto Research Chemicals (Abcam, Cambridge, United Kingdom) or Sigma-Aldrich (Irvine, United Kingdom) and either made up as stock solutions dissolved in aqueous media or in dimethyl sulfoxide (DMSO) (Sigma-Aldrich). Cyclohexanone and cyclohexanol were obtained from Sigma-Aldrich. Some preparations were pre-incubated in MPS containing 1–2 μM μ-conotoxin GIIIB (μCTX-GIIIB) (Sigma-Aldrich or Peptide Institute Inc., Osaka, Japan) to block muscle action potentials and contractions.

### Muscle force measurements

For muscle force measurements FDB preparations were pinned by their distal tendons to the base of a Sylgard-lined chamber and the proximal tendon was connected by 6/0 silk suture to an MLT0202 force transducer (ADInstruments, Oxford, United Kingdom). The preparations were bathed in 10 ml of MPS and the tibial nerve was aspirated into a glass suction electrode. Nerve stimuli (0.1–0.2 ms duration, nominally up to 10 V) were delivered via a DS2 stimulator (Digitimer, Welwyn Garden City, United Kingdom). Force recordings were captured and digitized at 1 kHz and analyzed via a Powerlab 26T interface using Labchart 7 software (ADInstruments) running on a Macintosh iMac computer, which was also used to trigger train-of-four (TOF) stimulation at 2 Hz and tetani (0.5–2 s duration) at 20–50 Hz.

### Ca^2+^ imaging

Flexor digitorum brevis preparations were incubated in normal MPS and the acetoxymethyl ester Fluo4-AM (Thermo Fisher Scientific, Waltham, MA, United States) was added to the bathing medium. Standard manufacturer’s recommended concentrations of Fluo4-AM (1–2 μM) did not produce sufficient labeling of muscle fibers. However, a relatively high concentration of Fluo4-AM (20 μM), suggested by Professor R. Robitaille (personal communication), usually achieved discernible loading of several FDB muscle fibers. After loading with Fluo-4, preparations were washed with MPS for 10–20 min and then mounted on the stage of the Olympus BX50WI microscope and imaged using an OptiMOS 2.1MP camera (Photometrics, Newcastle, United Kingdom) before or after adding μCTX-GIIIB and omethoate to the recording chamber. In some experiments, the Mg^2+^ concentration in the bathing medium was increased to 5 mM by adding MgCl_2_. Images were acquired at up to 100 frame per second (fps) using Micromanager public domain software (Edelstein et al., 2014), downloaded from https://micro-manager.org/. Images were analyzed using ImageJ^1^ or FiJI.^2^ Displacement of images in the focal plane were digitally compensated using the StackReg/TurboReg plugin.^3^ Muscle contractions in image sequences were measured using the Muscle Motion plugin for ImageJ/FiJI.^4^ Fluorescence image intensity was measured in image stacks of the junctional and extrajunctional regions of NMJs using the *Z*-axis Profile tool in ImageJ/FiJI, applied to rectangular or elliptical regions of interest (ROIs) covering the selected NMJs. Changes in fluorescence intensity were expressed either in arbitrary units of pixel gray level or as ΔF/F_*o*_, where ΔF represented the difference between the ROI intensity and the average intensity of the ROI before stimulation (F_*o*_). Surface plots of the ROIs were made using the inbuilt tool in ImageJ/FiJI.

### *Ex vivo* assay of synaptic degeneration

Synaptic degeneration was measured in cultures of isolated FDB and DL muscle preparations from 2 to 3 month old *thy1YFP16*:*Wld^S^* mice as described previously (Brown et al., 2015; Dissanayake et al., 2020). Briefly, muscles were pinned to dental wax slabs and placed in plastic vials containing bicarbonate-buffered MPS bubbled continuously with 95%O_2_/5% CO_2_. Either omethoate (100 μM) or a cocktail (DCOC), comprising dimethoate (1 mM) and cyclohexanone (1 mM), together with the principal metabolites omethoate (100 μM) and cyclohexanol (5 mM), was added to the incubation solutions to induce synaptic degeneration. These concentrations were chosen because they replicated the effects of dimethoate insecticide administration in acute experiments (Eddleston et al., 2012; Dissanayake et al., 2021a). In some experiments, the Mg^2+^ concentration in the bathing medium was increased to 5 mM by adding MgCl_2_. Vials were incubated in a water bath maintained at 32°C. After 24 h, motor endplates were counterstained by incubating in TRITC-α-bungarotoxin (5 μg/ml for 20 min) washed in MPS, fixed for 15 min in 4% paraformaldehyde/PBS, washed and imaged in a BioRad Radiance 2000 (Bio-Rad, Hemel Hempstead, United Kingdom) confocal microscope. Images were captured via a 40× oil immersion objective using inbuilt Lasersharp software. Five arbitrarily chosen microscope fields were captured and endplates were scored for occupancy by YFP-positive motor nerve terminals in each field. The scores were then averaged and plotted, with n equal to the number of muscles for the purpose of statistical evaluation.

### Electron microscopy

Flexor digitorum brevis muscles from acute experiments or following overnight incubation for assay of synaptic degeneration were fixed overnight in cold (4°C), 2% glutaraldehyde (Electron Microscopy Sciences, Hatfield, PA, United States) in 0.1 M sodium cacodylate (Sigma-Aldrich) buffer, pH 7.4. Fixed muscles were washed in buffer and transported on ice by courier from Edinburgh to Newcastle for further processing. Fiber bundles were post fixed in 1% osmium tetroxide (Agar Scientific, Stansted, United Kingdom) dehydrated in gradient acetone (Thermo Fisher Scientific) and impregnated with increasing concentrations of epoxy resin (TAAB Laboratories, Aldermaston, United Kingdom). Following several changes of 100% resin the fiber bundles were orientated to allow transverse or longitudinal sectioning, embedded in epoxy resin and polymerized overnight at 60°C. Survey sections (1 μm) of the muscle fibers were collected onto glass slides and stained with toluidine blue (TAAB Laboratories, Aldermaston, United Kingdom) to identify regions containing possible NMJs. Ultrathin (70 nm) sections of ROI were cut using a EM UC7 ultramicrotome (Leica Microsystems, Milton Keynes, United Kingdom) fitted with a diamond knife (Ultra 45°, Diatome, Hatfield, PA, United States), picked up on pioloform coated copper grids (Gilder Grids, Grantham, United Kingdom) stained with uranyl acetate (Agar Scientific, Stansted, United Kingdom) and lead citrate (Sigma-Aldrich) and imaged using either CM100 (Philips, Eindhoven, Netherlands) or HT7800 (Hitachi, Maidenhead, United Kingdom) transmission electron microscope with high-resolution digital image capture.

### Statistics and graphics

Data were analyzed and graphed using Excel (Microsoft, Reading, United Kingdom) and Prism 7 for Mac (GraphPad, La Jolla, CA, United States). ANOVA with Sidak’s *post hoc* tests were used to assess significance of mean differences.

### Software

The following commercial and public domain software was used: Office (Microsoft, Reading, United Kingdom); Prism (GraphPad, San Diego, CA, United States); Labchart (ADInstruments, Oxford, United Kingdom); Micromanager^5^; ImageJ (see text footnote 1); FiJi (see text footnote 2).

## Results

Our study utilized an anti-AChE model of excitotoxicity, based on the neuromuscular effects of the OP compound omethoate. This compound is the principal metabolite of dimethoate, an active ingredient in some agricultural insecticides (Eddleston et al., 2012; Eddleston, 2019; Dissanayake et al., 2021a). The present study represents an extension of preliminary findings based only on muscle function recordings that we reported previously (Dissanayake et al., 2021a). Summarizing, we reported in that study that omethoate inhibits muscle AChE with an IC50 of about 10 μM, while the IC50 for dimethoate is around 1 mM. Concentrations of 100–150 μM omethoate were sufficient to almost completely inhibit AChE in muscle homogenates and purified enzyme extracts. Adding omethoate (10–150 μM) to media bathing isolated nerve-muscle preparations gives rise to prolonged muscle force responses when the nerve supply is stimulated with short high-frequency trains (10–100 Hz for 0.5–2 s). These post-tetanic “aftercontractions” typically continued between 2 and 20 s after the end of the tetanic stimulus train but they sometimes lasted 30 s or longer. Further analysis showed that, after adding omethoate, aftercontractions developed following a latent period of about 30 min, became maximal after about 60 min and then declined in the continuing presence of omethoate over the following 2 h. Aftercontractions persisted when muscles were incubated in the Na_*V*_1.4 channel blocker μCTX-GIIIB. However, aftercontractions (but not tetanic responses) were strongly and selectively mitigated, in concentration-dependent fashion, by increasing the extracellular Mg^2+^ concentration and were maximally inhibited in 5 mM Mg^2+^. Imaging muscles in unstained preparations during and after stimulation showed that both the tetanic responses and the aftercontractions induced by omethoate most likely arose from direct effects on motor endplates. In a subsequent study (Dissanayake et al., 2021b) we found that omethoate caused prolonged, summating endplate currents (EPCs) and endplate potentials (EPPs).

The present study extended these observations and revealed possible links to synaptic pathology.

### Omethoate-induced aftercontraction has a long refractory period

**FIGURE 1:**
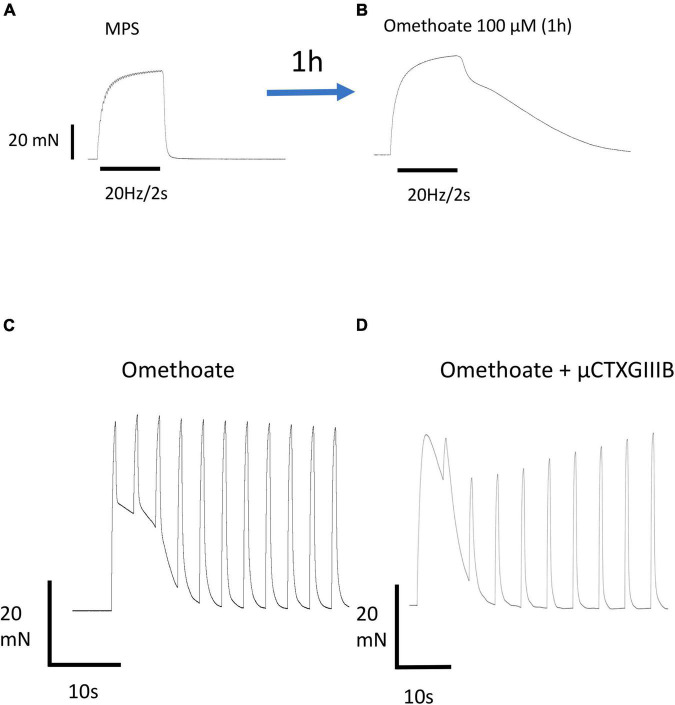
Omethoate induces prolonged aftercontractions. **(A)** Response of an isolated FDB muscle to repetitive stimulation at 20 Hz for 2 s. The partially fused tetanic response relaxes promptly at the end of the stimulus train. **(B)** Same preparation 1 h after incubation in 100 μM omethoate. A similar tetanic stimulus train produced a fused tetanus with a complex profile of prolonged relaxation (“aftercontraction”). **(C)** Aftercontractions have a long refractory period. Response of a different FDB preparation incubated for 1 h in 100 μM omethoate, stimulated at 40 Hz for 0.5 s approximately every 5 s. The first response comprises a brief tetanus followed by a prolonged aftercontraction. Successive tetanic stimulus trains caused force production during stimulation, summating with the relaxing phase of the aftercontraction due to the first train, but induced no aftercontractions. **(D)** Different preparation stimulated with a similar pattern of tetanic trains but co-incubated in 2 μM μCTX-GIIIB to block muscle action potentials and 100 μM omethoate for 1 h.

Figure 1A illustrates the typical force response of an isolated FDB nerve-muscle preparation to tetanic stimulation of its tibial nerve supply before, then approximately 1 h after adding omethoate (100 μM) to the bathing medium. The characteristic brisk onset and relaxation following the end of a tetanic stimulus in control MPS solution was transformed after adding omethoate to a complex profile of muscle force that diminished gradually as the muscle slowly relaxed (Figure 1B). Both responses were completely abolished by adding 5 μM α-bungarotoxin to the bathing medium, blocking ACh receptors (AChR) (data not shown). Aftercontractions were not exclusive to muscles treated with omethoate. Similar responses were observed after muscles were exposed to other classes of anti-AChE, including the carbamate AChE antagonist neostigmine (Hong and Chang, 1993; Dissanayake et al., 2021b) and the piperidine anti-AChE donepezil (Redman et al., submitted).

In addition to a relatively long latent period, omethoate-induced tetanic aftercontractions also showed a long refractory period. Figure 1C shows repeated responses to brief tetanic stimulation (40 Hz, 0.5 s) repeated every 5 s. The first train of stimuli produced a strong tetanic response with a slowly relaxing aftercontraction, like that also shown in Figure 1B. However, repeating these brief tetanic stimulus trains at intervals of 5 s produced active tension only during stimulation, with prompt relaxation and no aftercontractions at the end of the tetanic stimulus train. Rather, successive tetanic responses became superimposed on the slow decay of the aftercontraction produced by the first stimulus train. We did not attempt to quantify the refractory period accurately but recordings from several preparations consistently showed that aftercontractions in omethoate were partially or completely restored only after periods of 5–30 min rest between tetanic stimulus trains. Of note, both tetanic responses and refractory aftercontractions persisted after blocking muscle action potentials with μCTX-GIIIB, which abolishes nerve-evoked muscle contractile responses entirely in control MPS (Figure 1D).

**FIGURE 2:**
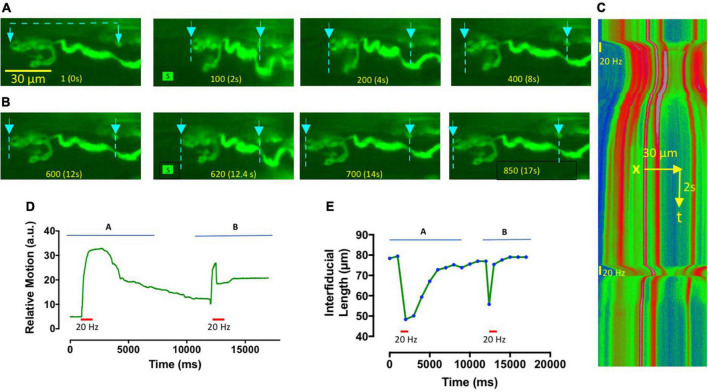
Omethoate-induced contractures and aftercontractions are localized to NMJs. **(A,B)** Image frames from Supplementary Video 1, showing an NMJ in TS muscle from a *thy1.2YFP16* mouse, after approximately 1 h incubation in omethoate (100 μM). Muscle Na_*V*_1.4 channels were also blocked by pre-incubation in μCTX-GIIIB. Two 20 Hz, 1 s stimulus trains (S)were delivered to this NMJ about 30 s apart. Frame numbers and corresponding times are indicated at the bottom of each frame. The first stimulus train **(A)** produced a powerful contracture and prolonged aftercontraction of the endplate, while the second tetanic stimulus **(B)** caused a rapidly adapting focal NMJ contraction for the duration of the stimulus train only (Compare with Figure 1C). Arrows and dotted lines show the locations of fluorescent points used as fiducial marks for analysis of endplate shortening and its kymographic display. **(C)** Kymograph of a line scan between the markers indicated on the video frames shown in panels **(A,B)**, pseudocoloured (Rainbow LUT in FiJI) in proportion to fluorescence intensity between the fiducial points indicated. The timings and duration of tetanic stimulation at 20 Hz are indicated by vertical bars. Note the prolonged shortening of the endplate region following the first tetanic response and the shorter period of endplate contracture in response to the second tetanic stimulus train. **(D)** Relative motion of the images obtained by analysis of the lowermost bouton using the Muscle Motion plugin for FiJI (arbitrary units of image intensity), further demonstrating that prolonged aftercontraction occurred in response to the first tetanus but the second produced a weaker, rapidly adapting tetanic response. **(E)** Plot of inter-fiducial length, indicative of endplate shortening. Symbols show measurements that were made on selected video frames at the time points indicated. Horizontal bars in panels **(D,E)** indicate the period of tetanic stimulation.

Imaging fluorescent NMJs demonstrated unequivocally that muscle aftercontractions were localized to the region of NMJs. Figures 2A,B shows an NMJ in a TS muscle preparation from the *thy1YFP16* transgenic mouse line, which expresses Yellow Fluorescent Protein in motor neurones and motor nerve terminals (Feng et al., 2000; Wong et al., 2009). The TS is a thin muscle, permitting good optical definition of fluorescent NMJs. Action potentials and active contractions in extrajunctional regions of the muscle were blocked in this preparation by pre-incubation in MPS containing μCTX-GIIIB, in addition to continuous incubation in 100 μM omethoate. After 1 h, brief tetanic stimulation (20 Hz, 0.5 s) of the innervating intercostal nerve gave rise to a long lasting, localized contracture of this and other NMJs in the same muscle. Figure 2C shows a kymograph plot comprising sequential frame-by frame line scans of fluorescence intensity between two fiducial points spanning the nerve terminal before and after the nerve supply was stimulated (see also Supplementary Video 1). Consistent with a long refractory period for aftercontractions, analysis of relative image motion and measurements of endplate length showed that a second 20 Hz stimulus strain delivered 20 s after the first caused a weakened endplate response during stimulation and no persistent aftercontraction (Figures 2D,E).

### Endplate aftercontractions are associated with persistent increases in endplate Ca^2+^

**FIGURE 3:**
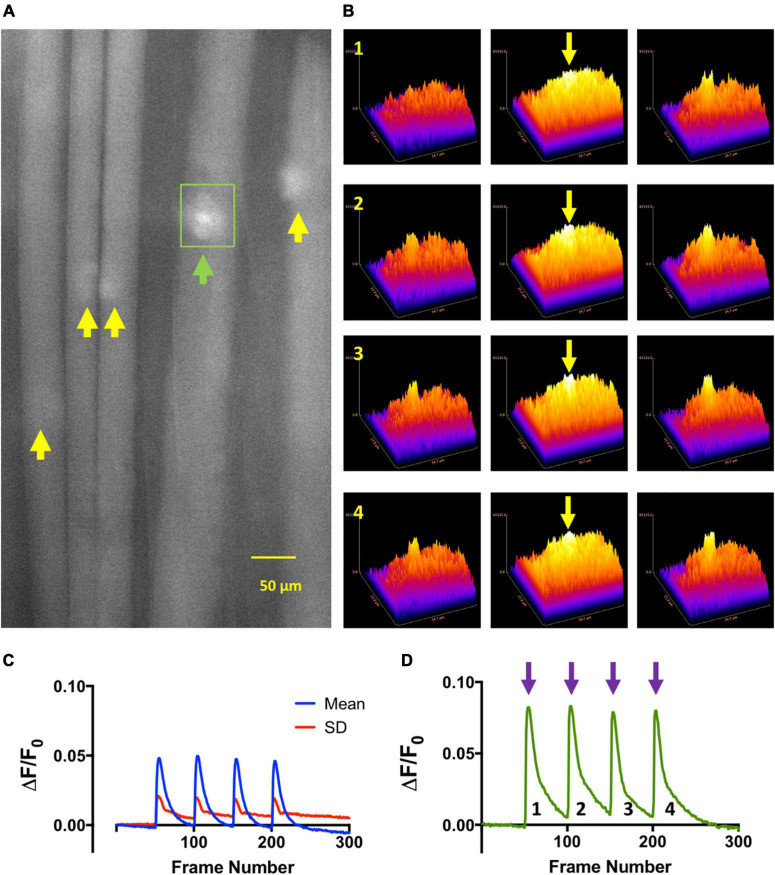
Train of Four (TOF) stimulation of NMJs in omethoate evokes local increases in endplate Ca^2+^. **(A)** Image of a mouse FDB muscle fibers loaded with the Ca^2+^ indicator Fluo-4AM and following incubation (>1 h) in 150 μM omethoate, at the peak of the twitch responses to each stimulus during train-of-four (TOF) stimulation at 2 Hz. Images were captured at 100 frames per second (fps). Fluo4 (Ca^2+^) signals were discernible only at the NMJs (arrows). **(B)** Three-dimensional surface plots of fluorescence from the local vicinity of the endplate indicated by the green arrow and rectangular region of interest outlined in panel **(A)** (see Supplementary Video 2). Rows 1–4 show endplate fluorescence associated with successive responses to TOF stimulation. The middle columns (yellow arrows) show images taken at the peak of the responses, the column on the left show images immediately before stimulation and the column on the right the responses 200 ms after the peak of the response. **(C)** Mean (blue line) and standard deviation (red) of fluorescence increases (ΔF/F_0_) in the endplate regions of the muscle fibers (*n* = 5 endplates) indicated by the arrows in panel **(A)**. **(D)** Increase in fluorescence (ΔF/F_0_) with each successive stimulus in the TOF for the endplate indicated by the green arrow in panel **(A)** and also corresponding to the surface plots shown in panel **(B)**.

Next we sought to establish directly the association between localized endplate contractures and intracellular Ca^2+^ at endplates, inferred but not directly demonstrated by previous studies of OP poisoning of endplate AChE (Leonard and Salpeter, 1979; Ferry and Cullen, 1991). We chose FDB for these experiments on account of the short length of its muscle fibers, which facilitated both loading with Fluo-4 and subsequent visualization of labeled endplate regions. In the absence of AChE inhibitors, low frequency (2 Hz) TOF stimulation produced time-locked increases in fluorescence running the entire length of muscle fibers with no obvious localization to NMJs. However, after incubation for more than 1 h in omethoate (150 μM) and μCTX-GIIIB, although variable in amplitude, TOF stimulation consistently produced flashes of Fluo-4 fluorescence localized to motor endplates (Figure 3 and Supplementary Video 2).

**FIGURE 4:**
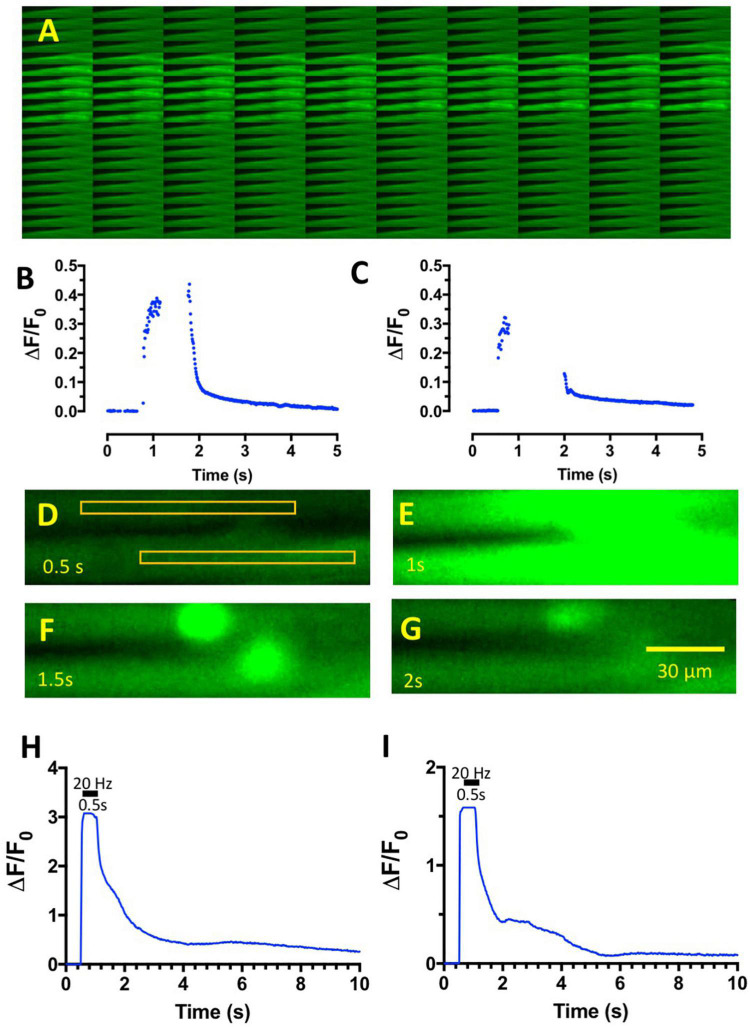
Tetanic stimulation of muscles incubated in omethoate causes complex endplate Ca^2+^ transients. **(A)** Montage of sequential images of an FDB muscle in control MPS solution during 1 s tetanic stimulation at 20 Hz. Bright region shows the period of increased Fluo-4 fluorescence, which was limited to the period of stimulation. Images captured at 100 fps. **(B)** Graph of fluorescence intensity (arbitrary units) against frame number (100 fps) of the images captured in panel **(A)**, showing the brisk onset and decay of intracellular Ca^2+^ associated with the tetanic stimulus train. Peak fluorescence data were discarded due to movement of the preparation in the image plane. **(C)** Similar data from a different preparation in control MPS. **(D–G)** Selected, sequential image frames (f25–f100: frames 25, 50, 75, and100 in Supplementary Video 3) from two FDB muscle fibers in a preparation loaded with Fluo-4 after incubation in 150 μM omethoate for 1 h then stimulated at 20 Hz for 0.5 s. Rectangles in panel **(D)** show the regions of interest (ROI) defined for measurement of average pixel intensity before **(D)**, during **(E)**, and after **(F,G)** tetanic stimulation. Note the persistence of endplate fluorescence in panel **(F)** and its subsequent slow decay. **(H,I)** Graphs of the upper **(H)** and lower **(I)** ROIs indicated in panel **(D)** over the duration of the recording shown in Supplementary Video 3. The period of stimulation (20 Hz, 0.5 s) is indicated by the horizontal bar. Images were captured at 50 fps. Note the complex profile of post-tetanic decay of fluorescence, due in part to prolonged, persistence fluorescence of the two NMJs contained in the image sequence.

It proved difficult to measure the profile of Fluo-4 fluorescence during tetanic stimulation in control MPS solutions, owing to marked displacement of the contracting muscle fibers from the image focal plane. Nevertheless, the responses illustrated in Figure 4A and graphed in Figures 4B,C showed, as expected, the brisk increase of intracellular Ca^2+^ levels at the onset of stimulation and its decay at the end of the tetanic stimulus train, visualized by the increase in Fluo-4 fluorescence. Ca^2+^-signals and muscle contractions in MPS were completely abolished after incubating muscles in the Na_*V*_1.4 channel blocker μCTX-GIIIB. However, following incubation in omethoate (100–150 μM) and μCTX-GIIIB (0.5 μM) for more than 1 h, short (0.5–1 s) trains of stimuli delivered at 20–50 Hz producing localized and prolonged and conspicuous fluorescence of the endplate region extending beyond the period of stimulation (Figures 4D–I; Supplementary Video 3). Moreover, the profile of fluorescence decay (Figures 4H,I) qualitatively resembled that of the muscle force measurements and endplate contractures shown in Figures 1, 2.

**FIGURE 5:**
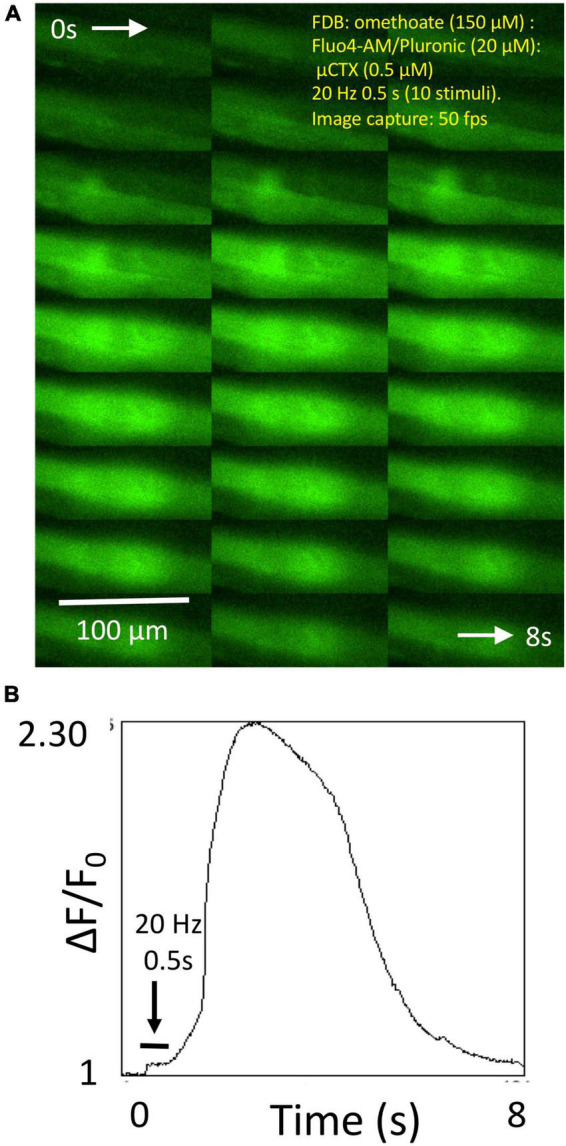
Omethoate induces “calcium bombs” in some muscle fibers. **(A)** Montage of sequential images of the endplate region of a group of FDB muscle fibers before during and after brief (20 Hz, 0.5 s) tetanic stimulation of the tibial nerve supply. **(B)** Plot of relative increase in fluorescence during the image sequence shown partly in panel **(A)** (complete sequence in Supplementary Video 4). Stimulation produced a relatively small increase in fluorescence during stimulation (horizontal bar) but which continued to increase, evidently regeneratively, after cessation of the tetanic stimulus train.

In other preparations, like the one shown in Figure 5, brief (0.5 s) tetanic stimulation caused a rise in endplate fluorescence that, surprisingly, continued to increase several fold after the end of the tetanic stimulus train, reaching a maximum level several hundred milliseconds later and subsequently showing complex decay characteristics over several seconds (Supplementary Video 4). We referred to these signals as “calcium bombs,” triggered by brief tetanic stimulation, on account of their apparent regenerative, explosive nature. These characteristics suggested the possibility of some form of a Ca-induced Ca-release (CICR) localized to NMJs (see Section “Discussion”).

### Prolonged Ca^2+^-transients are inhibited by millimolar increases in extracellular Mg^2+^

**FIGURE 6:**
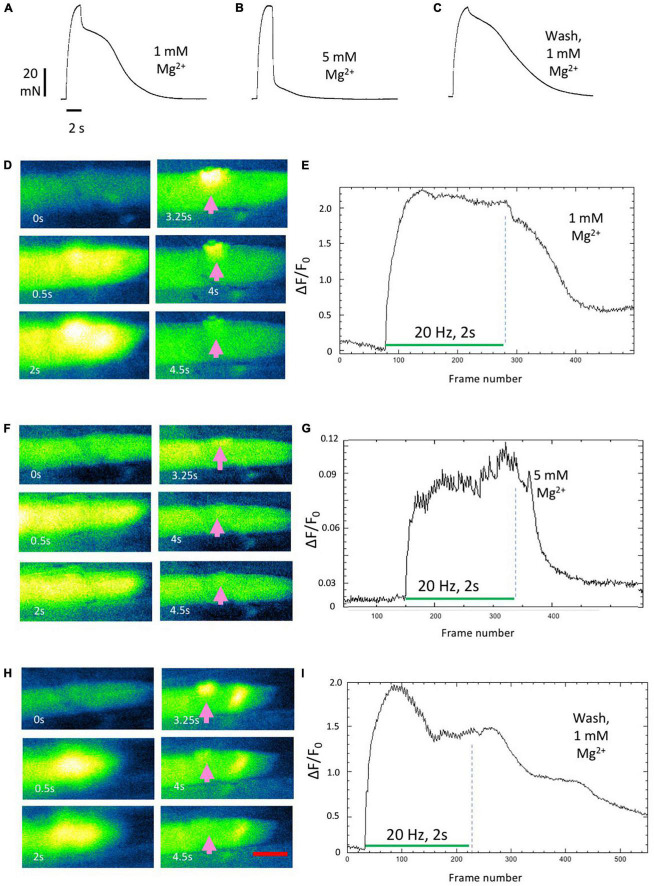
Aftercontractions of isolated mouse FDB muscles are reversibly mitigated by increasing extracellular Mg^2+^. **(A–C)** Isometric force recordings from an FDB muscle following approximately 1 h incubation in omethoate (150 μM). Increasing Mg^2+^ to 5 mM **(B)** abolished the aftercontraction, while leaving the response during the stimulus train unchanged. Aftercontraction immediately resumed after restoring Mg^2+^ to 1 mM **(C)**. **(D,E)** Fluo-4 images **(D)** and endplate ROI intensity plots in an FDB muscle during repetitive stimulation (20 Hz, 2 s), following 1 h incubation in omethoate. Intense endplate fluorescence (arrows) and contracture decayed slowly after the end of the stimulus train. **(F,G)** After increasing extracellular Mg^2+^ to 5 mM, stimulation provoked a weaker Ca^2+^ signal that decayed rapidly than in n 1 mM Mg^2+^. **(H,I)** Prolonged endplate Ca^2+^ fluorescence immediately returned after restoring Mg^2+^ to 1 mM. Appearance of an apparent ectopic region of fluorescence is unexplained but may have been due to an endplate in a different muscle fiber that drifted into the image plane. See Supplementary Videos 5–7. Calibration bar in panel **(E)** 20 μm.

As we reported previously (Dissanayake et al., 2021a), aftercontractions were reversibly abolished in 5 mM Mg^2+^. The recordings shown in Figure 6 replicated this finding. Aftercontractions were restored within less than 1 min after returning the preparation to normal MPS containing 1 mM Mg^2+^ (Figures 6A–C). Increasing the extracellular Mg^2+^ concentration to 5 mM also rapidly and reversibly mitigated associated, prolonged endplate Fluo-4 fluorescence (Figures 6D–I; Supplementary Videos 5–7). Specifically, tetanic stimulation in omethoate caused a prolonged localized increase in Fluo-4 fluorescence, with the endplate conspicuously highlighted in this instance for more than 2 s after the stimulus train had ceased (Figures 6D,E). The post-tetanic Ca^2+^ increase at the endplate was markedly attenuated and curtailed 30 min after adding 5 mM Mg^2+^ (Figures 6F,G). Endplate after-fluorescence and aftercontraction were immediately restored after returning Mg^2+^ concentration in the bathing medium to 1 mM (Figures 6H,I).

Quantitative replication of the effect of Mg^2+^ on tetanic Ca^2+^ signals evoked in individual Fluo-4 labeled fibers proved difficult, due to movement in the image plane caused by endplate regions undergoing contracture throughout the muscle. However, mitigating effects of 5 mM Mg^2+^, qualitatively similar to that shown in Figure 6, were replicated in two other preparations.

### Mg^2+^ also mitigates omethoate-induced synaptic degeneration *ex vivo*

Previous studies have shown that inhibiting AChE with OP compounds, or other circumstances in which AChE activity is compromised, triggers a focal myopathy, reported in some studies to trigger degeneration of motor nerve terminals (Leonard and Salpeter, 1979; Duxson and Vrbová, 1985; Hutchinson et al., 1993; Gomez et al., 2002). Since muscle aftercontractions and associated increases in endplate Ca^2+^ were inhibited by Mg^2+^ we therefore asked whether degeneration of NMJs induced by OP compounds would also be inhibited by a similar increase in extracellular [Mg^2+^]. We tested for this using an “*ex-vivo*” assay of neuromuscular synaptic degeneration (Brown et al., 2015; Di Stefano et al., 2015; Dissanayake et al., 2020). In the present experiments we utilized double homozygous *thy1YFP16*:*Wld^S^* mice whose axons and terminals were endogenously fluorescent due to expression of the YFP transgene in motor neurons (Feng et al., 2000; Wong et al., 2009; Brown et al., 2014). In these mouse strains “Wallerian” axonal and synaptic degeneration following axotomy are delayed for 3–10 days *in vivo* and 24–48 h *in vitro* (Mack et al., 2001; Gillingwater et al., 2002; Brown et al., 2015; Gilley et al., 2017). Thus, in preparations from *Wld^S^* mice, more than 80% of motor endplates are still occupied by motor nerve terminals 24–48 h after incubation in oxygenated MPS at 32°C. By contrast, fewer that 20% of motor nerve terminals remain intact after 24 h when preparations from control mice are similarly organ-cultured (Brown et al., 2015; Di Stefano et al., 2015; Dissanayake et al., 2020).

**FIGURE 7:**
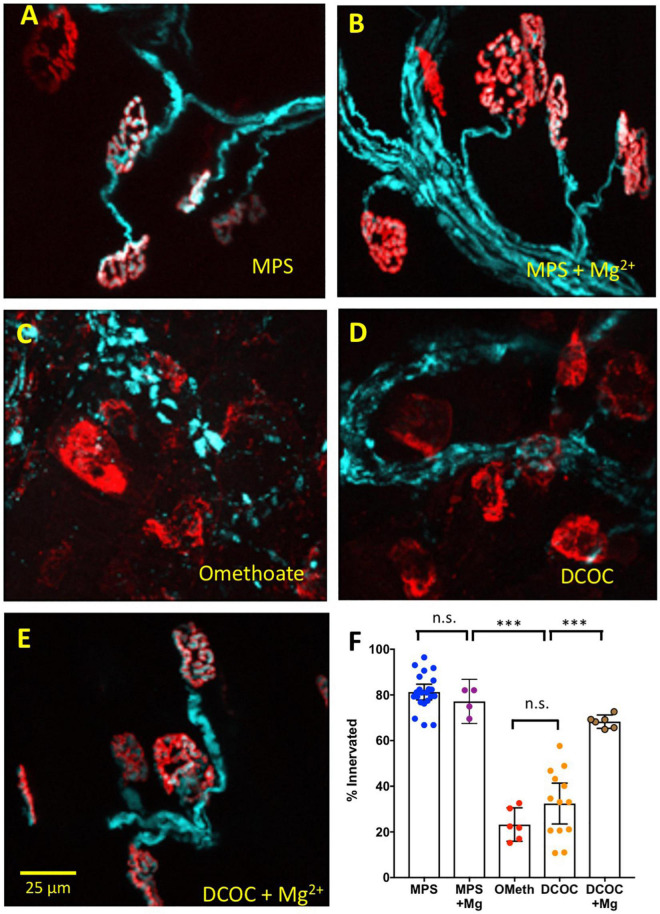
Mg^2+^ also mitigates NMJ degeneration. Data from *thy1.2YFP16:Wld*^S^** mouse lumbrical muscle preparations maintained for 24 h in oxygenated medium at 32°C. Pseudocoloured cyan fluorescence is from transgenic co-expression of YFP in motor neurons, including their axons and axon terminals; red counterstain is TRITC-α-BTC labeling of post-synaptic AChR. **(A)** Almost all NMJs remain innervated after 24 h culture in MPS; **(B)** 24 h incubation in MPS containing 5 mM Mg^2+^ did not change the protective effect of *Wld^S^*. **(C)** Incubation in MPS containing 100 μM omethoate overrode the *Wld^S^* protective phenotype and provoked degeneration and denervation of motor endplates at most NMJs. **(D)** Extensive degeneration and denervation was also found after 24 h incubation in a cocktail of dimethoate insecticide components dimethoate (1 mM), cyclohexanone (1 mM) and respective metabolites omethoate (100 μM) and cyclohexanol (5 mM). **(E)** Co-incubation in the DCOC cocktail with Mg^2+^ increased to 5 mM resulted in substantially less synaptic and intramuscular axonal degeneration. **(F)** Summary data showing percentage innervation of endplates after 24 h incubation in MPS, MPS with Mg^2+^ increased to 5 mM, 100 μM omethoate (OMeth), DCOC cocktail, DCOC with Mg^2+^ increased to 5 mM. Each point represents the mean percentage innervation from one muscle, based on measurements of 20–30 NMJs per muscle. Asterisks represent results of ANOVA (***P < 0.001; n.s.–*P* > 0.05) and *post hoc* Sidak’s test based on numbers (n) of muscles (*N* = 4–12 mice in each column).

In MPS containing 1 mM Mg^2+^, as previously, almost all motor nerve terminals in *thy1YFP16*-*Wld^S^* preparations remained fluorescent when cultured at 32°C for at least 24 h *ex vivo* (Figures 7A,B). However, incubation of these muscles either in omethoate (100 μM) alone, or in DCOC, a cocktail of components of a dimethoate-based agricultural insecticide and their metabolites (Eddleston et al., 2012), triggered extensive degeneration of motor nerve terminals, indicated by loss of YFP fluorescence from most NMJs. Fluorescent AChR staining with TRITC-α-BTX was largely preserved but we note that several endplates evidently lost or showed disruption of the “pretzel” organization of endplate AChR (Figures 7C,D). Electron microscopy (EM) supported the assessment of integrity of motor nerve terminals based on fluorescence microscopy. Supplementary Figure 1 shows electron micrographs of preserved motor nerve terminals in control preparations but degeneration of terminals after 24 h incubation in omethoate. In other EM sections there appeared to be extensive disruption of the muscle fibers in presumed regions of NMJs as well (data not shown), commensurate with the disrupted TRITC-α-BTX staining of some endplates observed with fluorescence microscopy and consistent with previous reports (Leonard and Salpeter, 1979; Ferry and Cullen, 1991).

Remarkably, in media containing DCOC, most motor nerve terminals and endplates were preserved when [Mg^2+^] was increased to 5 mM (Figure 7E). Specifically, 81.28 ± 7.8% of NMJs remained innervated (mean ± S.E.M; *n* = 22 muscles, *N* = 12 mice) in preparations bathed for 24 h in oxygenated MPS. In preparations co-incubated with omethoate only 23.25 ± 6.98% (*n* = 6 muscles, *N* = 4 mice) of fibers remained innervated (*P* < 0.0001, ANOVA, *post hoc* Sidak’s test *P* < 0.0001). In preparations incubated in DCOC, 32.45 ± 14.8% of NMJs remained innervated (*n* = 13 muscles, *N* = 8 mice; *P* < 0.0001, Sidak’s test). But after 24 h cultures in DCOC with [Mg^2+^] increased to 5 mM, degeneration of nerve terminals was substantially reduced: 68.33 ± 2.80% (*n* = 6 muscles, *N* = 4 mice) of NMJs remained innervated after 24 h in these cultures. While this remained 10–20% less than in control preparations (*P* = 0.024, Sidak’s test) it represented about two fold less degeneration compared with DCOC alone (*P* < 0.0001, Sidak’s test). There was no significant effect of 5 mM Mg^2+^ on the small amount of degeneration observed in control solutions (77.18 ± 6.08% endplates innervated *n* = 4 muscles, *N* = 4 mice; *P* > 0.05, Sidak’s test).

Thus, in sum, inhibiting AChE induced motor endplate contractures, prolonged endplate calcium transients, and promoted synaptic degeneration. The simplest hypothesis consistent with these observations is that pre-synaptic pathology, at least in this paradigm, is linked to excitotoxic triggers that bring about pathogenic changes in post-synaptic [Ca^2+^]. All three features were strongly mitigated by increasing extracellular Mg^2+^.

## Discussion

The present study produced three significant, novel findings. First, when AChE was strongly inhibited using omethoate, an OP toxin, tetanic stimulation of skeletal muscle through its nerve supply triggered substantial, transient increases in intracellular Ca^2+^ at motor endplates of muscle fibers. In some instances, the increases in endplate Ca^2+^ appeared to have regenerative (explosive) characteristics (“calcium bombs”). These outbursts extended over a region about 100–200 μm on either side of NMJs but they were most intense in the sub-synaptic region. Second, after 24 h exposure of nerve-muscle preparations to OP insecticide components and their metabolites, most neuromuscular synapses degenerated, even though motor nerve terminals in our organ culture assay are normally protected by expression of the Wld*^S^* protein. Third, both calcium bombs and enhanced synaptic degeneration were strongly mitigated by increasing extracellular Mg^2+^ to 5 mM. Previous studies have shown that modest increases in extracellular or systemic concentrations of Mg^2+^ restore characteristics of tetanic tension responses in isolated rodent preparations treated with OP compounds *in vitro* (Bradley, 1986) and in anesthetized pigs *in vivo* (Dissanayake et al., 2021a). Together, these observations support a working hypothesis that pathological increases in post-synaptic Ca^2+^ are both necessary and sufficient for induction and degeneration of NMJs by anti-AChE compounds. Secondly, Mg^2+^ therapies, or other treatments that mitigate post-synaptic Ca^2+^ loading should mitigate or prevent onset of pathological changes at synapses, pre-synaptically as well as post-synaptically.

### Mechanism and mitigation of “calcium bombs”

Direct observation of intracellular motor endplate Ca^2+^ transients following AChE inactivation with OP compounds has not been reported previously to our knowledge, although several previous studies have described localized contractures of NMJs, and attributed these by inference to Ca^2+^ loading of motor endplates (Leonard and Salpeter, 1979; Duxson and Vrbová, 1985; Burd and Ferry, 1987; Burd et al., 1989; Ferry and Cullen, 1991). Optical measurements of Ca^2+^-transients at motor endplates of skeletal muscle fibers are technically difficult to make, due to large and unpredictable movements of muscle fibers in the image plane during stimulation. Thus, the findings reported in the present paper are based on rather few, sometimes individual, observations. Nevertheless, our optical recordings were consistent with more robust, but indirect, muscle force measurements (Dissanayake et al., 2021a,b).

Remarkably, tetanic and post-tetanic responses in omethoate were resistant to block of muscle Na_*V*_1.4 channels with μCTX-GIIIB, which completely blocked muscle responses in control MPS solution. Thus, generation of muscle force through nerve stimulation after inhibiting AChE with omethoate did not require regenerative depolarization mediated by voltage-sensitive Na^+^ channels in muscle fiber membranes. Rather, under these conditions, forces (and Ca^2+^ signals) generated by neuromuscular transmission in the endplate region alone were sufficient to replicate tetanic force production by FDB muscle fibers in control solutions, which are normally completely blocked by μCTX-GIIIB. A plausible explanation for the force characteristics and some of the endplate Ca^2+^ transients is that they result from a localized form of regenerative, Ca^2+^-induced Ca^2+^ release (CICR). CICR was originally discovered in studies of skeletal muscle contraction but it is not normally thought to be important in the regulation of skeletal muscle force. By contrast, CICR is an important regulator of muscle contraction in cardiac myocytes and smooth muscle cells (Endo, 2009; Ríos, 2018). Of note, CICR is inhibited by increases in cytoplasmic Mg^2+^ concentration, possibly by competition with Ca^2+^ ions for binding to ryanodine receptors (RyR) in the terminal cisternae of the sarcoplasmic reticulum (Iaparov et al., 2021). Ca^2+^ release from sarcoplasmic stores in the endplate region could also occur via activation of IP3 receptors (Zayas et al., 2007; Zhu et al., 2011). However, the long refractory period for aftercontractions and Ca-signals, extending several seconds (see Figure 1), is difficult to reconcile with the relatively short refractory periods reported for CICR associated either with RyR or IP3 receptors (Maggi et al., 1994; Zemkova et al., 2004; Wang et al., 2014). The FDB muscle also contains a mixture of fast- and slow-twitch muscle fiber types (Banas et al., 2011) and there is evidence that CICR differs in fast- and slow-twitch muscles (Pagala and Taylor, 1998). It may therefore be interesting to establish whether persistent or regenerative endplate Ca^2+^ transients differ in muscle fibers of these different types after inhibition of their endplate AChE.

If endplate calcium-bombs were due to CICR, what would be the most likely source of the initial “trigger calcium”? Neuromuscular transmission at human and other vertebrate NMJs is mediated by nicotinic AChR, located post-synaptically in high density at the crests of the junctional folds at NMJs (Fertuck and Salpeter, 1976; Slater et al., 1992; Wood and Slater, 1995). Ca^2+^ permeability of AChR at rodent NMJs accounts for about 4% of the increase in cationic membrane permeability following binding of ACh but the increase in Ca^2+^ permeability at human NMJs is almost twice as great, amounting to about 7% of AChR current (Bregestovski et al., 1979; Villarroel and Sakmann, 1996; Ragozzino et al., 1998; Fucile et al., 2006). Thus, AChR currents are a plausible source of an initial rise in endplate “trigger” Ca^2+^, albeit that reduced AChE activity is evidently necessary to unmask it. Previous animal tissue studies have also shown that Ca^2+^ channel antagonists substantially reduced Ca^2+^-mediated responses of motor endplates (Wachtel, 1987; Moriconi et al., 2010; Piccari et al., 2011). In preliminary experiments we found that the L-type Ca^2+^ channel antagonists nimodipine or verapamil were also partially effective in reducing endplate contractures but at concentrations greater than those normally utilized therapeutically (Redman and Ribchester, unpublished observations). Verapamil was also effective in reducing endplate contractures produced by iontophoretic application of ACh to dissociated FDB muscle fiber preparations, from which AChE activity was stripped by enzymic digestion of the basal lamina (Ribchester, unpublished observations). Answering the question whether voltage-sensitive Ca^2+^ channels are involved in the generation of CICR at endplates may therefore benefit from further investigation of these observations.

However, other potential mechanisms could explain the complex profiles of post-tetanic decay of endplate Ca^2+^. Inhibiting AChE results in prolonged EPCs and summation of EPCs during repetitive stimulation, causing prolonged endplate depolarization (Dissanayake et al., 2021b). Moreover, increasing extracellular [Mg^2+^] reduces the conductance of the adult (ε-subunit) form of AChR at NMJs (Grassi and Degasperi, 2000; Piccari et al., 2011; Deflorio et al., 2012). Alternatively, or in addition, sustained post-synaptic Ca^2+^ signals and local contractures could also reflect prolonged, asynchronous ACh release from motor nerve terminals (Chang and Hong, 1986), which would also be a source of enhanced and prolonged endplate depolarization. We obtained some evidence for increased asynchronous ACh release in some of our intracellular recordings but not consistently. Specifically, reliable recording of large, tonic depolarization of the resting potential during tetanic stimulation when AChE is inhibited, even when Na_*V*_1.4 channels are blocked with μCTX-GIIIB, was difficult to distinguish from movement artifacts due to endplate contracture. The source of apparent increases in MEPP frequency during such recordings was also difficult to distinguish from the effects of mechanical damage to motor nerve terminals by the recording microelectrode when the endplate region was in motion (Dissanayake and Ribchester, unpublished observations).

Finally, Mg^2+^ increases the affinity of AChE for its inhibitors (Inestrosa et al., 1994; Ibrahim et al., 2009). However, we are inclined to rule out this property of AChE as the cause of Mg^2+^ sensitivity of aftercontractions or the underlying, prolonged Ca^2+^ signals, since AChE was already completely inhibited by omethoate at the concentrations used in the present experiments (Dissanayake et al., 2021a). Moreover, at the concentration of Mg^2+^ that was effective (5 mM) in mitigating aftercontractions and Ca-signals, there is no discernible effect on EPP decay time, comparable to that produced by either carbamate or OP AChE inhibitors (Dissanayake et al., 2021b).

In sum, further studies are required to establish whether Ca^2+^ flux through AChR triggers CICR in the endplate region or whether post-synaptic Ca^2+^ transients are linear or non-linear functions of membrane depolarization that map continuously to activation of AChR, Ca^2+^ channels or IP3 receptors. This may perhaps be resolved by optical recording of pre-synaptic Ca^2+^ or exocytosis in paralyzed muscles, eliminating movement artifacts (Tabares et al., 2007; Dittrich et al., 2018).

### Mitigation of synaptic degeneration

The data suggest that a concentration of Mg^2+^ that reduced prolonged Ca^2+^ transients also mitigated degeneration of NMJs in overnight organ cultures of *Wld^S^* mouse muscles, raising the possibility that Mg^2+^ might be utilized to delay progressive degeneration of synapses in other contexts. Grounds for such optimism include that NMJ contractures, like those mitigated by Mg^2+^, have been associated with the myopathic alterations in endplate morphology and subsequent degeneration of motor nerve terminals (Leonard and Salpeter, 1979; Duxson and Vrbová, 1985; Meshul et al., 1985; Ferry and Cullen, 1991). Degeneration of motor nerve terminals also occurs following direct injury to muscle fibers at motor endplates (Rich and Lichtman, 1989). Endplate pathology also occurs in several myasthenic syndromes associated with mutation in AChE or in slow-channel syndromes associated with mutations in AChR (Engel et al., 1982; Hutchinson et al., 1993; Gomez et al., 2002; Webster et al., 2013; McMacken et al., 2019). It has been suggested that as a consequence of the relatively high Ca^2+^ permeability of human junctional AChR, humans may be more vulnerable to myopathies induced by Ca^2+^ loading of their NMJs compared with rodent NMJs (Fucile et al., 2006). Ca^2+^ also accumulates abnormally in motor nerve terminals at NMJs in response to ischemia or oxidative stress in the SOD1 mouse model of ALS (David et al., 2007; Talbot et al., 2012). Testing efficacy of Mg^2+^ in other, relevant animal models of ALS could therefore be fruitful (Wong et al., 2009; Gilley et al., 2017).

### Conclusion and future prospects

The present study did not utilize either human tissue or more conventional animal models of synaptic pathology in ALS or FTD, such as the SOD1G93A or mutant TDP43 knock-in mouse models (Wong et al., 2009; White et al., 2018). Nevertheless, the findings reported here using an *in vitro* model of OP toxicity provide insight into processes that could also be critical for induction of synaptic pathology in several forms of disease in which synapses are vulnerable. Riluzole and edavorone are currently the only approved drugs for treatment of ALS and, thus far, prospective trials of other candidates have not been successful. Trials under way include the search for medications already established for treatment of other conditions that may be repurposed for treatment of ALS (Mehta et al., 2021). Mg^2+^ therapies have been reported in previous experimental studies and clinical trials focused on mitigation of OP toxicity (Bradley, 1986; Basher et al., 2013; Philomena et al., 2016; Vijayakumar et al., 2017; Jamshidi et al., 2018). The potential efficacy of magnesium has been considered previously in the context of nutrition or treatment of patients with ALS but clinical data from studies of MgSO_4_ in both contexts have not been decisive (Longnecker et al., 2000; Pamphlett et al., 2003; Fondell et al., 2013; Brvar et al., 2018; Aman et al., 2021; Kumar et al., 2022). However, administration of MgSO_4_ has proved clinically effective in treatment of eclampsia (Crowther, 1990; Fawcett et al., 1999; Duley et al., 2003). Further studies, including comparisons in animal or cellular *in vitro* models of ALS, may produce more insight into the mechanism of antagonism of Ca^2+^ transients by Mg^2+^ and whether reducing the magnitude of post-synaptic increases in intracellular Ca^2+^ may provide an alternative or additive treatment for conditions in which early signs of synaptic pathology are harbingers of neurodegenerative disease.

## Data availability statement

The raw data supporting the conclusions of this article will be made available by the authors, without undue reservation.

## Ethics statement

Ethical review and approval was not required for the animal study because isolated preparations from mice killed by UK Home Office Schedule 1 method were used, not requiring ethical review or approval.

## Author contributions

KD and RRe performed experiments, analyzed data, and helped write the manuscript. HM performed experiments, analyzed data, and reviewed the manuscript. ME acquired funding and contributed to study design and supervision. RRi acquired funding, designed study, performed and supervised experiments, analyzed data, and wrote the manuscript. All authors contributed to the article and approved the submitted version.

## Conflict of interest

The authors declare that the research was conducted in the absence of any commercial or financial relationships that could be construed as a potential conflict of interest.

## Publisher’s note

All claims expressed in this article are solely those of the authors and do not necessarily represent those of their affiliated organizations, or those of the publisher, the editors and the reviewers. Any product that may be evaluated in this article, or claim that may be made by its manufacturer, is not guaranteed or endorsed by the publisher.

## Footnotes

1
https://imagej.nih.gov/ij/

2
https://fiji.sc/

3
http://bigwww.epfl.ch/thevenaz/stackreg/

4
https://github.com/l-sala/MUSCLEMOTION

5
https://micro-manager.org/

## Supplementary material

Supplementary Figure 1 Electron micrographs of NMJs in isolated FDB muscles. **(A)** Control; **(B)** muscle incubated for 24 h in oxygenated, bicarbonate-buffered MPS; **(C)** muscle incubated for 1 h in omethoate (150 μM) then stimulated at 50 Hz during rapid fixation in 2% glutaraldehyde/0.1 M cacodylate buffer; **(D)** degenerating NMJ after 24 h incubation in oxygenated, bicarbonate-buffered MPS containing 100 μM omethoate.

Supplementary Video 1 Brief tetanic stimulation (20 Hz, 0.5 s; green box in lower left quadrant shows during stimulation only) of a YFP-labeled motor nerve terminal after incubation in omethoate and μCTX-GIIIB caused localized contracture of the endplate region and prolonged aftercontraction. A second stimulus train delivered a few seconds later produced a brisker endplate contraction during stimulation only with no overt aftercontraction.

Supplementary Video 2 Surface plots showing pulses of Fluo-4 fluorescence at an NMJ after incubation in 150 μM omethoate and 2 μM μCTX-GIIIB, in response to TOF stimulation at 2 Hz. See Figure 3.

Supplementary Video 3 Responses of two NMJs to brief tetanic stimulation (20 Hz, 0.5 s) in an isolated FDB muscle loaded with Fluo4-AM, after 1 h incubation in 150 μM omethoate and 0.5 μM μCTX-GIIIB. An explosive Ca^2+^ signal restricted to the endplate region decays slowly after the end of the stimulus train with notable persistence in the two endplate regions.

Supplementary Video 4 “Calcium bombs” in a cluster of muscle fibers in an isolated FDB muscle loaded with Fluo4-AM, after 1 h incubation in 150 μM omethoate and 0.5 μM μCTX-GIIIB. In this preparation, brief tetanic stimulation (20 Hz, 0.5 s) produced an initially weak Ca^+^ response which increased substantially in magnitude, apparently regeneratively, after the end of the tetanic stimulus train.

Supplementary Video 5 Response of an isolated FDB muscle loaded with Fluo4-AM, after 1 h incubation in 150 μM omethoate and 0.5 μM μCTX-GIIIB in MPS containing 1 mM Mg^2+^, during and after stimulation for 2 s at 20 Hz. Intense, prolonged fluorescence continued for several seconds after the stimulus train. The decay of fluorescence was complex.

Supplementary Video 6 Same muscle fiber as shown in Supplementary Video 4, 30 min after increasing extracellular [Mg^2+^] to 5 mM. A strong Ca^2+^ signal was produced during stimulation but the afterglow seen in Supplementary Video 4 was abolished.

Supplementary Video 7 Same muscle fiber as shown in Supplementary Videos 4 and 5, immediately after restoring [Mg^2+^] in the bathing medium to 1 mM. Prolonged fluorescence, like that observed in Supplementary Video 4 immediately returned. The cause of a second region of persistent intense fluorescence, not seen in Supplementary Videos 4 and 5, is unclear.
