# Supplementary figures and images for: “Calcium bombs” as harbingers of synaptic pathology and their mitigation by magnesium at murine neuromuscular junctions
Source: Front Mol Neurosci. 2022 Jul 26;15:937974. doi: 10.3389/fnmol.2022.937974 (PMC9361872; doi:10.3389/fnmol.2022.937974)

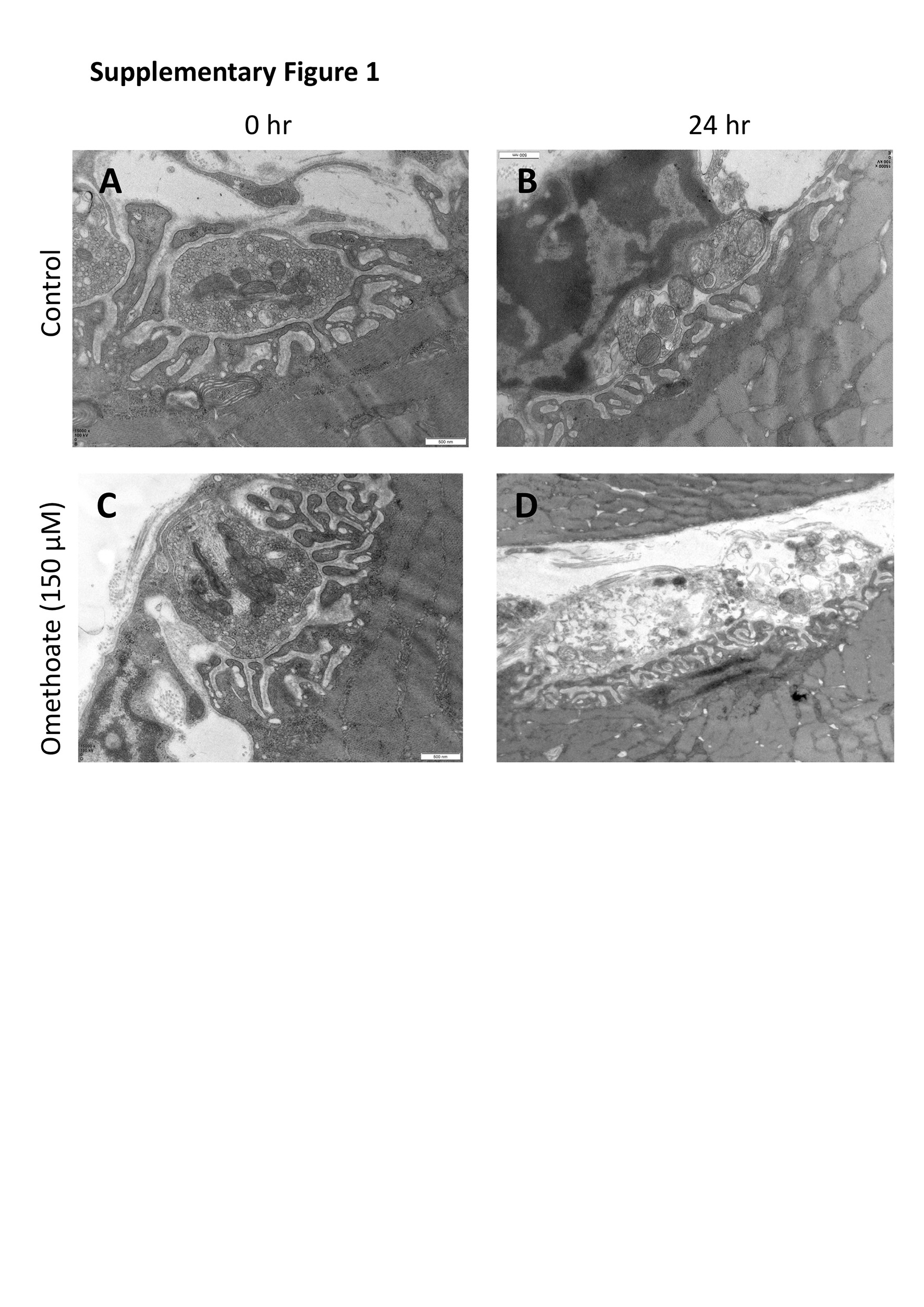

Supplement: Supplementary Figure 1 — Electron micrographs of NMJs in isolated FDB muscles. (A) Control; (B) muscle incubated for 24 h in oxygenated, bicarbonate-buffered MPS; (C) muscle incubated for 1 h in omethoate (150 μM) then stimulated at 50 Hz during rapid fixation in 2% glutaraldehyde/0.1 M cacodylate buffer; (D) degenerating NMJ after 24 h incubation in oxygenated, bicarbonate-buffered MPS containing 100 μM omethoate. [file Image_1.JPEG]
